# Anoctamin 1 antagonism potentiates conventional tocolytic-mediated relaxation of pregnant human uterine smooth muscle

**DOI:** 10.1186/s12576-021-00792-3

**Published:** 2021-02-22

**Authors:** Shunsuke Hyuga, Robert C. Parry, Jennifer Danielsson, Joy Vink, Xiao Wen Fu, Amy Wu, William Dan, Peter D. Yim, George Gallos

**Affiliations:** 1 Department of Anesthesiology, Columbia University College of Physicians and Surgeons, 622 W. 168th St. P&S Box 46, New York, NY 10032 USA; 2 Department of Obstetrics & Gynecology, Columbia University College of Physicians and Surgeons, Columbia University Medical Center, New York, NY USA

**Keywords:** ANO1, Calcium-activated chloride channel, TMEM16A, Uterine smooth muscle, Tocolytics

## Abstract

**Background:**

Currently available tocolytic agents are not effective treatment for preterm labor beyond 48 h. A major reason is the development of maternal side effects which preclude the maintenance of an effective steady-state drug concentration. One strategy that can mitigate these side effects is utilizing synergistic drug combinations to reduce the drug concentrations necessary to elicit a clinical effect. We have previously shown that three anoctamin 1 (ANO1) antagonists mediate potent relaxation of precontracted human uterine smooth muscle (USM). In this study, we aimed to determine whether a combination of sub-relaxatory doses of tocolytic drugs in current clinical use [the L-type voltage-gated calcium channel (VGCC) blocker, nifedipine (NIF); and the β_2_-adrenergic (β2AR) agonist, terbutaline (TRB)] will potentiate USM relaxation with two ANO1 antagonists [benzbromarone (BB) and MONNA (MN)].

**Objective:**

This study sought to examine the synergistic potency and mechanistic basis of two ANO1 antagonists with currently available tocolytic drugs. Functional endpoints assessed included relaxation of pre-contracting pregnant human USM tissue, inhibition of intracellular calcium release, and reduction of spontaneous transient inward current (STIC) recordings in human uterine smooth muscle cells.

**Methods:**

Human myometrial strips and primary human USM cells were used in organ bath and calcium flux experiments with different combinations of sub-threshold doses of ANO1 antagonists and terbutaline or nifedipine to determine if ANO1 antagonists potentiate tocolytic drugs.

**Results:**

The combination of sub-threshold doses of two ANO1 antagonists and current tocolytic drugs demonstrate a significant degree of synergy to relax human pregnant USM compared to the effects achieved when these drugs are administered individually.

**Conclusion:**

A combination of sub-threshold doses of VGCC blocker and β2AR agonist with ANO1 antagonists potentiates relaxation of oxytocin-induced contractility and calcium flux in human USM ex vivo. Our findings may serve as a foundation for novel tocolytic drug combinations.

## Introduction

Preterm birth (PTB) remains a major obstetric crisis affecting one in every ten births and is the leading cause of neonatal death in the US [1, 2]. Etiologies of PTB vary; however, one of the most common causes of PTB is spontaneous preterm labor (PTL). Although there have been recent advances in the prevention of PTB such as progesterone therapy [3], it requires early initiation and is not indicated in the treatment of spontaneous PTL. Currently available tocolytics [nifedipine (NIF), terbutaline (TRB), and indomethacin] are complicated by maternal side effects that preclude the maintenance of effective steady-state drug concentrations contributing to clinical inefficacy beyond 48 h [4]. These side effects include hypotension with NIF as well as tachycardia and tachyphylaxis with TRB. Given its limited efficacy, the pharmacological armamentarium for PTL requires novel treatment strategies that reduce maternal side effects. In other diseases, there is growing interest in repurposing drugs that possess synergistic effects in combination to utilize lower doses of each medication to avoid side effects known to occur after chronic, high-dose treatment [5–10]. This manuscript looks to determine if conventional tocolytics already employed in clinical use may be repurposed into drug combinations that may be clinically useful. Successful combinations of drug classes often address a common final functional pathway; therefore, it is necessary to understand the mechanisms of both uterine excitability and quiescence.

Uterine smooth muscle cell activation leading to coordinated uterine contractions is a complex, multi-faceted process that involves alterations in USM cell contractile agonist receptors, changes in ion channel composition, and increases in gap junctions, which are channels that transmit action potentials between cells. These changes allow the uterus to transform from a quiescent muscle bed with a low intrinsic excitability into a more responsive contractile USM phenotype that displays high intrinsic excitability [11–15]. It is understood that a critical component of this process involves interactions between several USM cell ion channels to promote changes in membrane potential. In particular, when the net effect of USM cell ion channel effects allows for a shift to a more depolarized potential, it allows for recruitment and activation of voltage-dependent ion channels (e.g. voltage-gated calcium channels [VGCCs]) to augment intracellular calcium entry and promote a widespread action potential wave into adjoining myometrial cells. However, it should be emphasized that since these channels are voltage-gated, they require a depolarizing threshold be met to activate them. As term gestation and labor approaches, resting membrane potential in USM will depolarize closer to the threshold, and the calcium activated chloride channel (CaCC) family is proposed to be important for this antecedent depolarizing drive. CaCC currents have long been recognized as important modulators of smooth muscle excitability [16–19]. With regard to excito-mechanical coupling, Jones et al. demonstrated that CaCC are activated by calcium entry via voltage-gated calcium channels (VGCC), highlighting a relationship between CaCC and VGCC in both spontaneous and oxytocin-stimulated contractions in myometrium [16]. In the USM cell, CaCC activation leads to an enhanced outward chloride current to further depolarize the uterine smooth muscle cell membrane and thereby increase excitability. In this context, CaCC channel-mediated depolarization may assist to promote VGCC’s (L-type) activation for enhancing action potential generation.

Another important modulator of uterine smooth muscle excitability is the β2-adrenoreceptor (β2AR). The β2AR is a G protein-coupled receptor, which activates adenylyl cyclase, increases cAMP and activates protein kinase A (PKA) [20, 21]. PKA ultimately inhibits phosphorylation of myosin light chain to indirectly promote relaxation of smooth muscle fibers. Interestingly, there is some evidence that agonism at the β2AR leads to activation of Ca^2+^/calmodulin-dependent protein kinase II (CaMKII) [22–24]. Furthermore, it has been suggested that CaMKII inhibits CaCCs [25], and recently, it was shown that CaMKII specifically inhibits anoctamin 1 (ANO1), a well-established CaCC [26–28]. In this context, we postulate that activation of the β2AR may, therefore, affect the activity of the CaCC ANO1 in labor via CaMKII-mediated inhibition.

Our laboratory has previously established the functional importance of ANO1 in promoting human and murine USM contractility. ANO1 is expressed in human myometrial tissue throughout gestation, and the blockade of these channels inhibits oxytocin-induced human and murine USM contractility and calcium release [29, 30]. Furthermore, we have reported on the comparative potency of several ANO1 antagonists (including benzbromarone [BB] and MONNA [MN]) on pregnant human USM relaxation, contraction frequency reduction, inhibition of intracellular calcium release and membrane hyperpolarization [31]. Given the mechanistic interplay between CaCCs and the conventional tocolytic targets VGCC and β2AR, we hypothesize that combination of ANO1 antagonists with these tocolytics may allow for improved efficacy with lower doses, potentially helping circumvent deleterious side effects. This study aims to establish if antagonism of ANO1 acts synergistically with low doses of the L-type VGCC blocker NIF or the β2AR agonist TRB to inhibit oxytocin-induced human USM contractions and calcium flux.

## Methods

### Reagents/chemicals

All reagents were purchased from Sigma-Aldrich (St. Louis, Missouri) unless stated otherwise. BB, MN and NIF were dissolved in dimethyl sulfoxide (DMSO). TRB was dissolved in double distilled water (ddH_2_O).

### Human USM specimens

In accordance with the Institutional Review Board (IRB)-approved protocol (#AAAL4005), de-identified fresh human uterine tissue was obtained from the superior margin of the uterine incision performed following elective cesarean deliveries at 38–40 weeks of gestation. All tissue samples were from non-laboring patients. In all cases, the tissue was immediately placed in cold, sterile Hank’s balanced salt solution (HBSS) on ice. The tissue samples were subsequently processed to establish smooth muscle strips for organ bath studies.

### Cell culture

Pregnant human USM cells were harvested from tissue samples mentioned above for use in culture using an enzymatic cellular dissociation kit (Worthington, Lakewood, NJ). The cells were seeded into a 75-cm^2^ culture flask and grown in smooth muscle basal medium-2 (SmBm-2) with manufacturer’s recommended additives (Lonza, Walkersville, MD, USA).

### Calcium flux studies

All calcium studies were performed using the ratiometric fluorescent calcium indicator Fura-2 (Calbiochem, Billerica, MA, USA) as previously described [32]. Human USM cells were grown to 100% confluence in 96-well black-walled clear-bottom plates and were used at between passages 4 and 8. Cells were washed with modified HBSS (concentration in mM: NaCl, 137.9; KCl, 5.3; CaCl_2_, 2.0; MgSO_4_, 1.0; HEPES, 2.4; and glucose, 5.5; pH–7.4). The cells were then loaded with 100 μL of 5 μM Fura-2 AM in a humidified 37 °C incubator (95% air/5% CO_2_) for 30 min. Cells were washed again with HBSS and then incubated in HBSS for 20 min to allow for de-esterification of the indicator.

#### Preliminary single-drug dose–response studies

The cells were pretreated (10 min) with NIF, TRB (each 0.1 nM—100 μM), or the vehicle. Then, the cells were exposed to oxytocin 1 μM to induce G protein-coupled receptor (GPCR)-mediated calcium release. The fluorescence was measured in real time at 37 °C using a Flex Station 3 (Molecular Devices) at excitation wavelengths of 340 and 380 nm, emission wavelength of 510 nm, and a cutoff filter of 495 nm. Fluorescence values were reported as F–F_0_ and calcium flux was calculated as follows: [ΔF = F_peak_(340 nm)/F_peak_(380 nm)—F_0_(340 nm)/F_0_(380 nm)], where F_0_ represents baseline fluorescence and F_peak_ represents peak fluorescence.

#### Combination drug potentiation studies

Each sub-threshold concentration was derived in section A above except for that of BB and MN, which were established in a previous study [31]. Cells were pretreated for 10 min with either a single agent (the ANO1 blockers BB (1 µM) or MN (10 µM); or NIF (0.001 µM); or TRB (0.001 µM) or a combination of BB (1 µM) or MN (10 µM) and NIF (0.001 µM) or TRB (0.001 μM), or the vehicle. Then, the cells were exposed to oxytocin 1 µM to induce GPCR-mediated calcium release. The fluorescence was measured as described above.

### Functional organ bath force recordings

Freshly obtained late gestation myometrium samples were finely dissected into 4 × 6 × 15 mm strips and attached inferiorly to a fixed tissue hook in a 16-mL organ bath (Radnoti Glass Technology, Monrovia, CA) and superiorly to a Grass FT03 force transducer (Grass Telefactor, (Biopac Systems, Goleta, CA). The Grass FT03 force transducer was used to continuously record the tensile force of the strip. The uterine strips were equilibrated under an applied tension of 2.5 g for 1 h in a modified Krebs–Henseleit buffer (concentration in mM: sodium chloride [NaCl], 112.0; potassium chloride [KCl], 5.0; calcium chloride [CaCl_2_], 2.5; magnesium sulfate [MgSO_4_], 1.2; sodium bicarbonate [NaHCO_3_], 25.0; monosodium phosphate [NaH_2_PO_4_],1.0; and D-glucose, 11.5). The buffer was warmed to 37 °C, replaced every 15 min for 1 h, and continuously bubbled with 95% O_2_/5% CO_2_. The muscle-force of intact uterine strips then was measured in response to exogenous oxytocin (0.5 μM) over 60 min. To limit variability in contraction frequency and decay over time, an EC_85_ (85% effective concentration) dose of oxytocin (0.5 μM) was employed to stimulate contractions (as shown by other investigators) [33].

#### Preliminary single-drug dose–response studies to pharmacologically characterize NIF and TRB

Following contractile stimulation with the addition of oxytocin, the uterine tissue strips were allowed to equilibrate at the increased baseline contractility for 60 min, and then, they were treated with NIF or TRB (each 0.1 nM—100 μM) or vehicle (0.1% DMSO or ddH_2_O, respectively) as a control. Following the addition of these drugs, the tensile force was analyzed over the next 60 min. For each strip, integral force (g·s) of the tocolytic treatment period was normalized to the integral force of its respective equilibrated baseline. Treated strips were compared to time-matched and vehicle-treated controls.

#### Combination drug potentiation studies

Following contractile stimulation with oxytocin, the uterine tissue strips were allowed to equilibrate at the increased baseline contractility for 60 min, and then we evaluated the effect of low-dose cotreatments with the test drugs. Each drug concentration was derived in section A above except for that of BB and MN, which were established in a previous study [31]. Specifically, we assessed the relaxation induced by treatment with either the vehicle (0.1% DMSO final concentration), BB (5 μM), MN (25 μM), NIF (0.01 μM), TRB (0.01 μM), or cotreatment with BB (5 μM) or MN (25 μM) and NIF (0.01 μM) or TRB (0.01 μM). Following the addition of these regimens, the tensile force was analyzed over the next 60 min. Changes in force were measured by normalizing the integral force of the treatment period to the integral force of each strip’s equilibrated oxytocin-induced contractility.

#### Effect of CaMKII inhibition on ANO1 inhibitor and β-agonist potentiation.

Following contractile stimulation with oxytocin, the uterine tissue strips were allowed to equilibrate at the increased baseline contractility for 60 min. CaMKII Inhibitor XII (Calbiochem, Billerica, MA, USA) was used to target CaMKII, with IC_50_ of 0.063 μM for CaMKII and IC_50_ > 10 μM for other tested kinases [34]. Contracting strips were treated with vehicle (0.1% DMSO) or CaMKII Inhibitor XII (1 μM) for another 60 min. Then, we assessed relaxation induced by treatment with either vehicle (0.1% DMSO) or cotreatment of BB (5 μM) and TRB (0.01 μM). Following the addition of these regimens, the tensile force was analyzed over the next 60 min. Changes in force were measured by normalizing the integral force of the treatment period to the integral force of each strip’s equilibrated oxytocin-induced contractility.

### Patch clamp electrophysiological studies in human USM cells treated with terbutaline

To assess membrane potential currents relevant to the physiology of contractility, we performed whole-cell patch clamp studies. Human USM was digested with collagenase type IV at 37 °C for 5–10 min. Released USM cells were plated on poly10 L-Lysine 12 mm coverslips (BD, San Jose, CA), coated with 0.5 mg/ml collagen Type I (Sigma) and incubated for another 1–3 days. For whole-cell recordings, coverslips were transferred to a 0.5 mL chamber on the stage of an inverted microscope (Nikon). Membrane currents were recorded using whole-cell configuration. The extracellular solution contained (in mM) 130.0 NaCl, 5.5 tetraethylammonium chloride (TEA-Cl), 2.2 CaCl_2_, 1.0 MgCl_2_, 10.0 HEPES, 10.0 Glucose, pH adjusted to 7.35 with NaOH. The pipette solution contained (in mM): 75.0 CsCl, 64.0 Cs-gluconate, 1.0 MgCl_2_, 10.0 HEPES, 3.0 Na_2_ATP, pH adjusted to pH 7.3 with CsOH. Whole-cell currents were recorded using Axopatch 200B coupled to a 1322A digitizer. The patch pipette had a resistance of 3–5 MΩ. All recordings were performed at room temperature. Criteria for quantifying spontaneous transient inward currents (STICs) included currents with an amplitude twice the baseline as detected with pCLAMP10 and analyzed by Origin 8 software. A voltage ramp from − 60 mV to 40 mV was performed and STICs were recorded to generate a voltage-current relationship. To demonstrate the effect of ANO1-specific blockade on STIC activity, we employed a functional TMEM16A (ANO1) antibody (to elicit complete and specific blockade) in live cell measurements of STICs (as previously published [35]). For these studies, cells were incubated for 3 h (in 37 °C, 5% CO2) with either TMEM16A antibody (Abcam ab53213, 1:5 dilution) or with the vehicle diluent used in the antibody as a control (0.1% sodium azide, 1% BSA, 50 mM Tris, pH 7.6), after which STIC activity was measured. In parallel studies, the impact of TRB on STICs were analyzed at a holding potential of − 60 mV to correlate with the natural resting membrane potential of USM [36]. Briefly, following recording of spontaneous STICs, three different concentrations (10 µM, 50 µM, or 100 µM) of TRB were applied, and STICs were measured. TRB used was reconstituted from stock solutions to final working concentration on the day of the experiment.

### Statistical analysis

Unless otherwise stated, the data were analyzed using a one-way analysis of variance (ANOVA) with the Bonferroni correction for multiple comparisons or unpaired two-tailed t tests (for comparing two groups) where appropriate. The data are illustrated using GraphPad software, and are expressed as the mean ± standard error (SE) and a *p* < 0.05 was considered statistically significant.

## Results

### NIF and TRB dose-dependently reduces both peak intracellular calcium response and oxytocin-induced contractile force

**Fig. 1:**
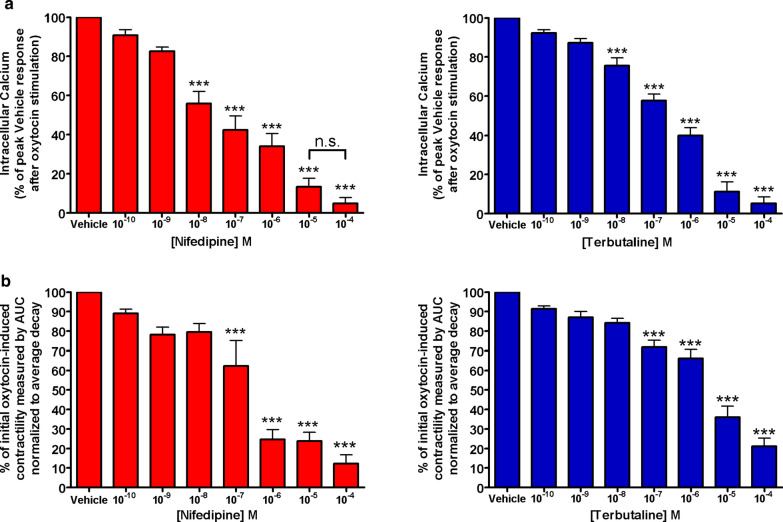
Pharmacological doses of terbutaline and nifedipine mediate reductions in oxytocin-induced intracellular calcium flux and contractile force in a dose-dependent manner. **a** Determination of subtherapeutic concentration for nifedipine and terbutaline. Initial dose–response experiments performed to assess significant reductions in intracellular calcium flux mediated by nifedipine (left) and terbutaline (right) demonstrated non-statistical reductions in intracellular calcium at doses up to 10^–9^ M. (***p < 0.001, n = 10). **b** Determination of subtherapeutic concentration for nifedipine and terbutaline. Initial dose–response experiments performed to assess significant reductions in contractility mediated by nifedipine (left) and terbutaline (right) demonstrated non-statistical reductions in force at doses up to 10^–8^ M (***p < 0.001, n = 38 samples from 8 different patients)

The preliminary dose–response Fura-2 calcium assays assessing the potential reduction in contractility by NIF or TRB demonstrated dose-dependent reductions in calcium flux. (Fig. 1a). The preliminary dose–response organ bath study assessing the potential reduction in contractility by NIF or TRB demonstrated dose-dependent reductions in force (Fig. 1b). However, NIF and TRB had insignificant reductions in calcium flux at 0.001 μM and in integral force at 0.01 μM. Further experiments used these established subtherapeutic doses of NIF and TRB.

### Low-dose NIF and ANO1 blockade: intracellular calcium response and oxytocin-induced contractile force are significantly reduced by a combination of the two treatments

**Fig. 2:**
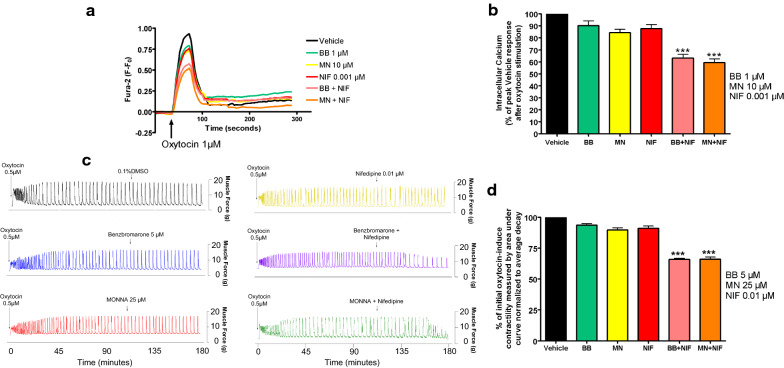
Combination of low dose L-type voltage-gated calcium channel blockade with ANO-1 blockade enhances relaxation of human uterine smooth muscle in terms of intracellular calcium flux and contractile force. **a** Representative fura-2 calcium tracings demonstrating that pretreatment with BB or MN in combination with NIF attenuates increase in intracellular calcium induced with oxytocin. BB = benzbromarone, MN = MONNA, NIF = nifedipine. **b** Average peak of intracellular calcium induced with oxytocin 1 µM after pretreatment demonstrating significant attenuation of intracellular calcium with low dose NIF + BB or NIF + MN versus the BB, MN, or NIF alone groups (***p < 0.001; for BB, MN, and NIF, n = 10 each; for NIF + BB or NIF + MN, n = 14 each). **c** Representative force tracings showing the enhanced potency of combining low dose NIF and BB (purple tracing) or MN (green tracing) on both frequency and force of oxytocin-induced contractions compared to single low dose treatments (BB 5 μM: blue, MN 25 μM: red, NIF 0.01 μM: yellow) or vehicle control (0.1%DMSO: black tracing). **d** Compiled data illustrate percentage of integral force (g*sec) compared to baseline oxytocin-induced contractility between treatment groups. Results were normalized to the control and reported as mean ± SEM. We observed statistically significant potentiation of relaxation with low dose NIF + BB/MN versus control (***p < 0.001; n = 64 samples from 9 different patients)

We sought to determine if low-dose NIF would act in combination with ANO1 blockade to synergistically reduce oxytocin-induced elevation of intracellular calcium in human USM cells and oxytocin-induced contractility in ex vivo human USM strips. In experiments using human USM cells, the representative tracings of Fura-2 fluorescence versus time, with 1 μM oxytocin added, is shown in Fig. 2a. We observed a statistically significant reduction of the response with low-dose NIF + BB/MN versus treatment with BB, MN, or NIF alone (***p < 0.001, Fig. 2b). Compared to control, calcium flux reductions were 6.6% for BB alone, 15.7% for MN alone, and 12.1% for NIF alone. Meanwhile, calcium flux reductions were 36.6% for NIF + BB and 40.4% for NIF + MN. Notably, the combinations have greater reductions than the sum of the reductions of the individual drugs.

In experiments using ex vivo human USM strips, the representative force tracings show the enhanced potency of combination low-dose NIF with BB/MN on both amplitude and integral force (measured by area under curve) of oxytocin-induced contractions compared to single low-dose treatments or the vehicle (Fig. 2c). We observed significantly greater relaxation with low-dose NIF + BB/MN versus treatment with BB, MN, or NIF alone (***p < 0.001, Fig. 2d). Compared to control, contractility reductions were 6.4% for BB alone, 10.3% for MN alone, and 8.8% for NIF alone. Meanwhile, contractility reductions were 34.1% for NIF + BB and 33.9% for NIF + MN. As the combinations again have greater reduction than the sum of the reductions of the individual drugs, we believe there is mechanistic synergy between the L-type VGCC blocker NIF and ANO antagonists at subtherapeutic doses.

### Low-dose TRB and ANO1 blockade: intracellular calcium response and oxytocin-induced contractile force are significantly reduced by a combination of the two treatments

**Fig. 3:**
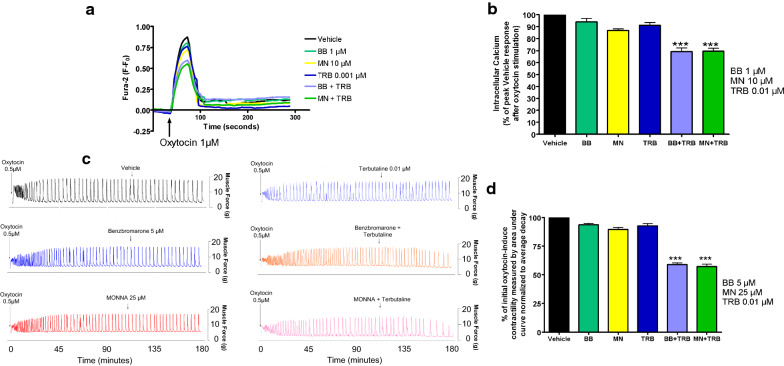
Combination of β2 adrenergic receptor agonist with ANO-1 blockade enhances relaxation of human uterine smooth muscle in terms of intracellular calcium flux and contractile force. **a** Representative fura-2 calcium tracings demonstrating that pretreatment with BB or MN in combination with TRB attenuates increase in intracellular calcium induced with oxytocin. BB = benzbromarone, MN = MONNA, TRB = terbutaline. **b** Average peak of intracellular calcium induced with oxytocin 1 µM after pretreatment demonstrating significant attenuation of intracellular calcium with low dose TRB + BB or TRB + MN versus the BB, MN, or TRB alone groups (***p < 0.001; for BB, MN, and TRB, n = 10 each; for TRB + BB or TRB + MN; n = 14 each). **c** Representative force tracings showing the enhanced potency of combining low dose TRB and BB (orange tracing) or MN (pink tracing) on both frequency and force of oxytocin-induced contractions compared to single low dose treatments (BB 5 μM: blue, MN 25 μM: red, TRB 0.01 μM: light blue) or vehicle control (black tracing). **d** Compiled data illustrate percentage of integral force (g*sec) compared to baseline oxytocin-induced contractility between treatment groups. Results were normalized to the control and reported as mean ± SEM. We observed statistically significant potentiation of relaxation with low dose TRB + BB/MN versus control (***p < 0.001; n = 64 samples from 9 different patients)

Next, we sought to determine if low-dose TRB would act in combination with ANO1 blockade to synergistically reduce oxytocin-induced elevation of intracellular calcium in human USM cells and oxytocin-induced contractility in ex vivo human USM strips. In experiments using human USM cells, the representative tracings of Fura-2 fluorescence versus time, with 1 μM oxytocin added, is shown in Fig. 3a. We observed a statistically significant reduction of the response with low-dose TRB + BB/MN versus treatment with BB, MN, or TRB alone (***p < 0.001, Fig. 3b). Compared to control, calcium flux reductions were 6.1% for BB alone, 13.2% for MN alone, and 8.9% for TRB alone. Meanwhile, calcium flux reductions were 30.7% for TRB + BB and 30.4% for TRB + MN. Notably, the combinations have significantly greater reduction than the sum of the reductions of the individual drugs.

In experiments using ex vivo human USM strips, the representative force tracings of the oxytocin-induced contractions compared to single low-dose treatments or the vehicle (Fig. 3c) are shown. We observed significantly greater relaxation with low-dose TRB + BB/MN versus treatment with BB, MN, or TRB alone (***p < 0.001, Fig. 3d). Compared to control, contractility reductions were 6.4% for BB alone, 10.3% for MN alone, and 7.2% for TRB alone. Meanwhile, contractility reductions were 41.2% for TRB + BB and 42.9% for TRB + MN. As the combinations again have greater reductions than the sum of the reductions of the individual drugs, we believe there is mechanistic synergy between the β2AR agonist TRB and ANO antagonists at subtherapeutic doses.

### Terbutaline inhibits STICs on human USM cells in a dose-dependent manner

**Fig. 4:**
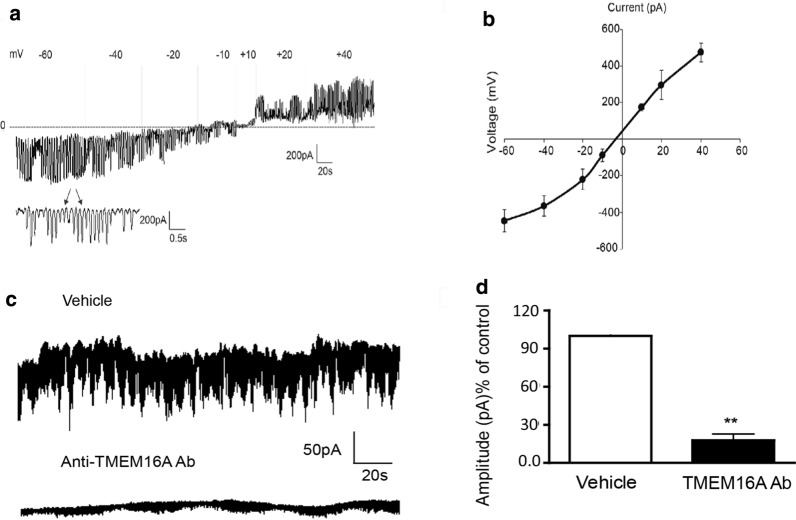
Spontaneous transient inward currents (STICs) recorded in human primary uterine cultured cells are ANO1-mediated. **a** Representative tracings of spontaneous transient inward currents (STICs) recorded at different holding potentials (Vm). Inset are STICs displayed at a higher resolution time scale. **b** Compiled I/V relationship (mean peak amplitude of STICs vs. voltage holding potential) demonstrates the observed reverse potential for STICs is − 7.5 ± 10 mV (n = 4), which closely approximates the predicted reversal potential for chloride (E_Cl_ of − 13.8 mV). **c** Representative whole-cell membrane STICs recordings from human USM cells perfused with either vehicle buffer (upper tracing) or with a TMEM16A (ANO1) antibody for use on live cells (lower tracing). Abrogation of STIC activity was observed (14 of 20 cells patched) following incubation with antibody compared to incubation with vehicle (3 of 20 patched cells). **d** Comparison of STIC amplitude between vehicle-treated or TMEM16A (ANO1) antibody-treated cells. There was significant attenuation of STIC amplitude by 82 ± 6% compared with vehicle controls (**p < 0.01)

While the mechanism of synergy between inhibition of VGCCs and CaCCs is established, the mechanism of synergy between the β2AR agonist TRB and ANO1 antagonists is not well understood. Therefore, we sought to elucidate the mechanism by which low-dose TRB and ANO1 antagonists potentiate relaxation of human USM. We have previously demonstrated that ANO1 mediates STICs in murine USM [29]. We now sought to confirm that ANO1 mediates STICs in human USM to investigate TRB’s effect on STICs and thus ANO1 activity. STICs recorded from cultured human USM cells under a holding potential − 60 mV demonstrate a continuously random inward current (Fig. 4a) with a spontaneous rhythm (Fig. 4a, inset). The average amplitude and frequency of the spontaneous rhythm current is − 100.2 ± 22 pA (range: − 400 to 76 pA) and 18.2 ± 3 Hz (range 60–4 Hz), respectively (n = 36, calculated events 1260). The average measurement of this current reached a peak with a mean rise time of 10.3 ± 3 ms and displays a single exponential function decay time of 18.8 ± 6 ms (n = 36, calculated events 1260). Figure 4b plots STICs at ramping holding potentials. The average E_Cl_ was determined to be − 7.5 ± 1.0 (mV) (n = 4), close to the predicted chloride reversal potential (E_Cl_) of − 13.8 mV [37]. The current at potentials more negative than − 7.5 mV were inward, and STICs changed direction from inward to outward at potentials more positive than the E_Cl_ reversal potential. This data are consistent with STICs occurring through a chloride channel such as ANO1. To corroborate these results and definitively show STIC activity is dependent on ANO1 (TMEM16A) channel activity—we also employed a TMEM16A antibody targeting ANO1 channels that specifically blocks channel activity in live cells. Antibody-mediated blockade of TMEM16A (ANO-1) led to a functional loss of STIC activity compared with vehicle-treated cells (Fig. 4c, d). We found abrogation of STIC activity in 14 of the 20 cells following incubation with antibody compared to diminished STIC activity in 3 of 20 patched cells following incubation with vehicle. There was significant attenuation of amplitude by 82 ± 6% (p < 0.01) compared to the control group. Taken together, these data prove that STICs are ANO1-mediated in human USM cells.

**Fig. 5:**
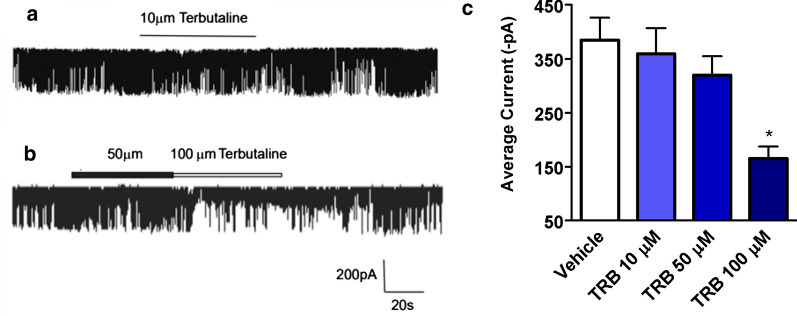
Terbutaline inhibits spontaneous transient inward currents (STICs) recorded from human primary uterine cultured cells. **a** Representative whole-cell recordings of STICs in response to terbutaline 10 µM. At holding voltages − 60 mV potential, the average current was − 359.8 ± 97 pA (events 251 ± 99, n = 5), compared to control − 385.2 ± 82 pA (events 247 ± 67, n = 6). **b** Representative whole-cell recordings of STICs in response to terbutaline 50 µM or 100 µM. With perfusion of terbutaline 50 µM at holding voltage − 60 mV, the average current was − 320 ± 69.5 pA (events 181 ± 45, n = 5). At terbutaline 100 µM, the average current was reduced to − 165.6 ± 44.1pA (events 108 ± 35, n = 6). **c** Bar graph depicting the effects of different concentrations of terbutaline on average current of STICs. At terbutaline 100 µM, the difference was statistically different from control (*p < 0.05)

We next found that TRB rapidly attenuates baseline STIC activity in a dose-dependent manner. At holding voltage − 60 mV potential, the average amplitude with 10 µM TRB was − 359.8 ± 97 pA (events 251 ± 99, n = 5), compared to control − 385.2 ± 82 pA (events 247 ± 67, n = 6, Fig. 5a). With perfusion of TRB 50 µM, the average amplitude was − 320 ± 69.5 pA (events 181 ± 45, n = 5). At TRB 100 µM, the average amplitude was − 165.6 ± 44.1pA (events 108 ± 35, n = 5), which was significantly different compared to control group (*p < 0.05, n = 5, Fig. 5b). Figure 5c, a compiles bar graph representing the results of different concentrations (10 µM, 50 µM or 100 µM) of TRB on amplitudes of STICs, illustrates 100 µM TRB reduced STICs in human USM cells compared to control groups (*p < 0.05, n = 6). These data demonstrate that TRB decreases STICs, which is evidence that TRB inhibits ANO1 functional activity.

### CaMKII mediates the synergistic relaxation from the combination of β2AR agonist with ANO1 blockade in precontracted human USM strips

**Fig. 6:**
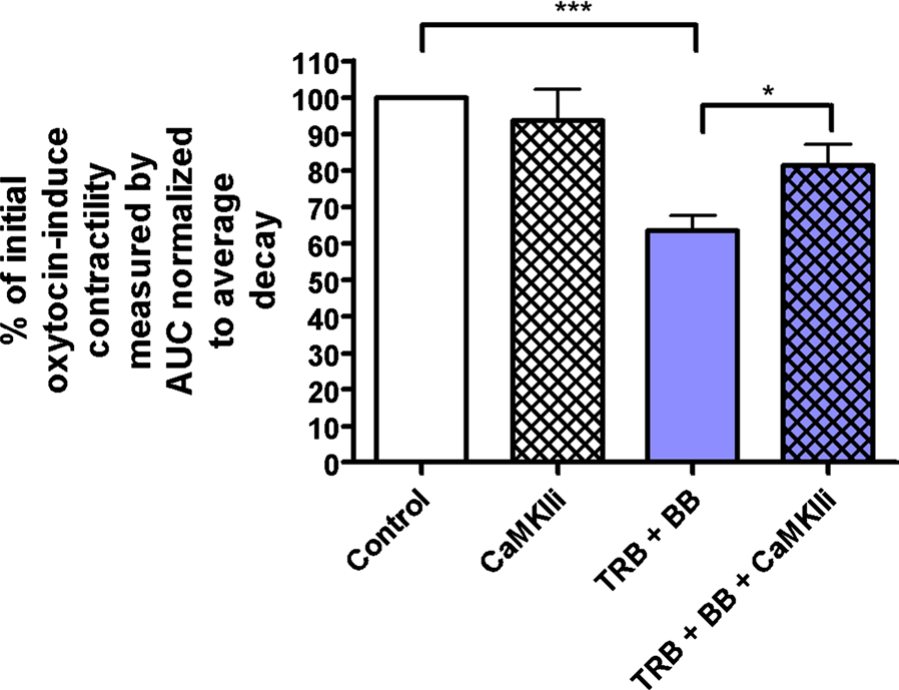
CaMKII mediates the synergistic relaxation from the combination of β2AR agonist with ANO1 blockade in precontracted human USM strips. Compiled data illustrate percentage of reduction in integral force (g*sec) from baseline oxytocin-induced contractility between treatment groups. Results were normalized to the control and reported as mean ± SEM. We observed no change with CaMKII Inhibitor XII (1 µM) alone, and again see statistically significant potentiation of relaxation with low-dose TRB + BB versus control. Interestingly, pretreatment with CaMKII Inhibitor XII mitigated the relaxation associated with the combination of TRB and BB (*p < 0.05, ***p < 0.001; n = 48 from 7 different patients)

After we determined that TRB attenuates ANO1 activity in STICs studies, we sought to determine if CaMKII mediates this effect. CaMKII Inhibitor XII was developed by Asano et al. to specifically inhibit CaMKII [34] and has been validated in a number of other studies [38, 39]. We utilized CaMKII Inhibitor XII to investigate the role of CaMKII in the relaxation effects seen between TRB and BB on oxytocin-induced contractility in ex vivo human USM strips (Fig. 6). CaMKII Inhibitor XII alone had no effect on contractility, while low-dose TRB + BB yielded significant potentiation of relaxation (p < 0.001). However, pretreatment with CaMKII Inhibitor XII mitigated the relaxation associated with the combination of TRB and BB (p < 0.05). Taken together, these data suggest CaMKII plays a significant role in the synergistic interaction between the β2AR agonist TRB with ANO1 inhibitor BB.

## Discussion

Preterm labor and delivery remain a major healthcare challenge. Notwithstanding the recent advances in preterm birth prevention, the management of spontaneous preterm labor with currently available tocolytics (magnesium sulfate, NIF, TRB, and indomethacin) is ineffective beyond 48 h [4]. Chronic use of these drugs in parturients can lead to increased maternal morbidity related to the side effects of these tocolytic agents [40–42]. One therapeutic strategy worth considering, is whether combinational therapy may facilitate enhanced clinical efficacy. This study highlights how ANO1 antagonists function synergistically with conventional VGCC blockers or β2AR agonists at low doses to inhibit oxytocin-induced contractions and calcium flux in human USM cells. The findings in this study suggest synergistic effects between ANO1 antagonists and these conventional tocolytic agents.

Antagonists of the calcium-activated chloride channel ANO1 are a class of novel candidates for combination therapy with the current tocolytic drug NIF (a VGCC blocker). Since ANO1 blockade results in hyperpolarization [31], it reduces the capacity to reach the voltage gating threshold of VGCC in USM cells. Other mechanisms may additionally contribute. For example, the non-inactivating Ca^2+^ current through the VGCC has been demonstrated to be of a lower magnitude at more negative potentials in the range of resting membrane potential [43]. As ANO1 blockade causes hyperpolarization, the combined effects of ANO1 inhibitors and NIF limiting the non-inactivating Ca^2+^ current may be partly responsible for the synergy demonstrated between these agents (Fig. 2). Our findings are consistent with CaCC channel-mediated depolarization complementing VGCC activation for enhancing action potential generation, with inhibitors of VGCC and ANO1 together creating an opposite, synergistic effect with lower doses of each antagonist.

In addition, ANO1 antagonists are novel candidates for combination therapy with the current tocolytic drug TRB, a β2 agonist. We observed a statistically significant potentiation of relaxation with low-dose TRB + BB/MN versus treatment with BB, MN, or TRB alone in functional organ bath and calcium flux studies (Fig. 3). As the mechanistic interplay between β2 agonism and ANO1 antagonism are less obvious, we further probed the mechanisms involved. TRB’s attenuation of STICs suggests TRB can independently influence ANO1 activity (Figs. 4 and 5). The attenuation of the synergistic relaxation by CaMKII inhibition confirmed CaMKII is a key mediator in the interplay between β2 agonism and ANO1 antagonism (Fig. 6). Our data demonstrate that the tocolytic TRB and ANO1 antagonists can work synergistically to relax USM with low doses of each drug as a novel potential therapy to treat PTL.

It should be noted, there is existing controversy regarding β-agonism and CaMKII activation. It has been shown that non-selective β-agonism activates CaMKII and that β2AR overexpression causes increased CaMKII activity [22–24]. However, cardiology studies posit that the β1AR but not the β2AR activate CaMKII-based off data from Mangmool et al. [44]. Their data use β1-KO and β2-KO mice to assess the role of both receptors in CaMKII activation from non-selective β-agonism with isoproterenol. While the β2-KO mouse did have higher CaMKII activation, the β1-KO mouse also had about a twofold, statistically significant increase in p-CaMKII. In addition, differential tissue expression of β-adrenoceptors is well known—in particular, the heart has much lower expression of the β2AR compared to the uterus; the β1AR is the predominant subtype in the heart, while the β2AR is the predominant subtype in the uterus [45–47]. Our data are consistent with TRB-induced inhibition of ANO1 in a mechanism at least partially mediated by CaMKII.

Our study has several strengths and limitations. We rigorously demonstrated the potential for ANO1 antagonists to be paired with current tocolytics NIF and TRB at low doses, which may help avoid maternal side effects associated with these drugs. Additionally, the combination regimen may allow for dosage to be increased from the low doses used in combination up to standard doses to maintain efficacy as tocolytic effects begin to wane after 48 h. Finally, our investigation of the mechanisms of synergy between ANO1 antagonists and TRB and NIF helps describe important interactions between ANO1 and other procontractile pathways in uterine smooth muscle. The use of pharmacologic inhibitors of ANO1 compared to more specific genetic techniques does limit the specificity for the observed effects attributed to ANO1. However, the consistency of the effect observed with two separate pharmacologic inhibitors for ANO1 helps address these concerns. In addition, while ex vivo uterine strip models are the gold standard for initial drug investigations to target PTL, combination regimens still require in vivo studies before having an opportunity to advance to any clinical studies.

In conclusion, the combination of low-dose ANO1 antagonists and low-dose current tocolytic drugs act synergistically together. Our study provides evidence that drug development including ANO1 antagonists holds promise as a potential tocolytic drug for managing spontaneous preterm labor.

## Data Availability

Data may be made available upon request to George Gallos.
